# Autologous Bone Marrow Stem Cell Transplantation in Liver Cirrhosis after Correcting Nutritional Anomalies, A Controlled Clinical Study 

**DOI:** 10.22074/cellj.2019.6108

**Published:** 2019-06-15

**Authors:** Abbas Esmaeilzadeh, Homeira Ommati, Mohammad Mahdi Kooshyar, Lida Jarahi, Kambiz Akhavan Rezayat, Samaneh Saberi, Massoud Vosough, Ali Ghassemi

**Affiliations:** 1 Department of Internal Medicine, Faculty of Medicine, Mashhad University of Medical Sciences, Gastroenterology and Hepatology Research Center, Mashhad, Iran; 2 Department of Hematology and Oncology, Faculty of Medicine, Mashhad University of Medical Sciences, Mashhad, Iran; 3 Department of Community Medicine, Faculty of Medicine, Mashhad University of Medical Sciences, Mashhad, Iran; 4 HPGC Group, Medical Biotechnology Department, Biotechnology Research Center, Pasteur Institute of Iran, Tehran, Iran; 5 Department of Regenerative Biomedicine, Cell Science Research Center, Royan Institute for Stem Cell Biology and Technology, ACECR, Tehran, Iran; 6 Department of Stem Cells and Developmental Biology, Cell Science Research Center, Royan Institute for Stem Cell Biology and Technology, ACECR, Tehran, Iran; 7 Department of Pediatric Hematology and Oncology, Faculty of Medicine, Mashhad University of Medical Sciences, Mashhad, Iran

**Keywords:** Bone Marrow Stem Cells, Cell Therapy, Cirrhosis, Regenerative Medicine

## Abstract

**Objective:**

Liver transplantation is the gold standard approach for decompensated liver cirrhosis. In recent years, stem cell therapy has raised hopes that adjusting some clinical and laboratory parameters could lead to successful treatments for this disease. Cirrhotic patients may have multiple systemic abnormalities in peripheral blood and irregular cell populations in bone marrow (BM). Correcting these abnormalities before BM aspiration may improve the effectiveness of cell-based therapy of liver cirrhosis.

**Materials and Methods:**

In this controlled clinical trial study, 20 patients with decompensated liver cirrhosis were enrolled. Patients were randomly assigned to control and experimental groups. Blood samples were obtained to measure vitamin B12, folate, serum iron, total iron bonding capacity (TIBC) and ferritin before any intervention. Furthermore, the iron storage and fibrosis level in BM biopsies, as well as the percentage of different cell populations, were evaluated. Prior to cell isolation for transplantation, we performed palliative supplement therapy followed by a correction of nutritional deficiencies. Mononuclear cells (MNCs) were then isolated from BM aspirates and transfused through peripheral vein in patients in the experimental group. The model of end-stage liver disease (MELD) score, The international normalized ratio (INR), serum albumin and bilirubin levels were assessed at 0 (baseline), 3 and 6 months after cell transplantation.

**Results:**

The MELD score (P=0.0001), INR (P=0.012), bilirubin (P<0.0001) and total albumin (P<0.0001) levels improved significantly in the experimental group after cell transplantation compared to the baseline and control groups. Moreover, the increase in serum albumin levels of patients in the experimental group was statistically significant 6 months after transplantation.

**Conclusion:**

We have successfully improved the conditions of preparing -BM-derived stem cells for transplantation. Although these cells are relatively safe and have been shown to improve some clinical signs and symptoms temporarily, there need to be more basic studies regarding the preparation steps for effective clinical use (Registration number: IRCT2014091919217N1).

## Introduction

Liver cirrhosis is one of the most common causes of death in the world and imposes huge financial burden (1, 2). Currently, the only available treatment for decompensated liver cirrhosis is orthotopic liver transplantation (OLT), which is limited to parameters such as the number of donated organs from cadavers or living donors, the high costs associated with both the procedure and the follow- up care, as well as post operation complications due to lifelong immunosuppression (3). However, recently researchers have focused on safe alternative possibilities to restore liver mass and function through stem cell therapy (4-7). 

In 1999, Petersen et al. (8) showed that hematopoietic stem cells can contribute to liver regeneration. In 2000, Theise et al. (9) reported that hematopoietic stem cells successfully transformed into hepatocytes and cholangiocytes. They found Y-chromosome-positive hepatocyte-like cells in the liver of female recipients who had received male BM stem cells. Likewise, in another study in 2002, researchers showed that hematopoietic stem cells in both peripheral blood and BM could differentiate into hepatocytes and other epithelial cells (10). Since 1956, various hematologic disorders have been treated by bone marrow (BM) transplantation and different related clinical studies have been carried out using BM transplantation (11). Given these findings, hematopoietic stem cells may be a promising source for cell therapy in liver cirrhosis. In addition, various hematologic abnormalities, such as nutritional deficiencies, are secondary to liver cirrhosis and directly affect the population of BM-derived cells (12, 13). 

To the best of our knowledge, at this point there is no study that has evaluated the efficacy of hematopoietic stem cell transplantation after correcting nutritional abnormalities in cirrhotic patients. Therefore, this clinical study was conducted to evaluate the efficiency of BM-mononuclear cell (MNC) transplantation through peripheral vein in cirrhotic patients after supplement treatment. Following cell transplantation, we also assessed whether the improved cells enhanced the results of stem cell therapy in cirrhotic patients. 

In studies of liver diseases, a commonly used value is the model for end-stage liver disease (MELD), which is a scoring system for quantifying the severity of chronic liver diseases. To predict the survival rate, MELD uses the patient’s values for serum bilirubin, serum creatinine, and international normalized ratio (INR) for prothrombin time (PT). Because the result (in seconds) for a PT performed on a normal individual will vary according to the type of analytical system, INR has been developed to normalize the results. 

In the present clinical trial, we show a significant decrease in patient MELD scores at 3 and 6 months’ post- cell transplantation. 

## Materials and Methods

### Patient criteria and treatment 

This controlled clinical trial study conducted at Mashhad University of Medical Science. Twenty patients were admitted at the "Research Center of Transplantation", Mashhad, Iran, from September 2014 to June 2015. All patients were diagnosed with liver cirrhosis, based on clinical, laboratory, radiologic and endoscopic data, and were all on a waiting list for liver transplantation. All patients received their regular medical treatment during the study. The exclusion criteria were refractory ascites, positive HIV antibody, primary sclerosing cholangitis (PSC), hepatocellular carcinoma (HCC), and portal and/or hepatic vein thrombosis.

Patients were randomly distributed into two groups, the experimental (n=10) and the control groups (n=10). Randomization was performed to reduce any possible bias and to adjust the study arms. In BM aspiration, our preferred site was the posterior iliac crest. Only the experimental group received intravenous infusion of autologous BM-derived MNCs. All the infusions were performed using veins in upper extremity. 

Prior to the infusion of autologous BM-derived stem cells, the serum levels of vitamin B12, folate, iron, TIBC and ferritin were measured in each participant. In addition, the BM aspirates were analyzed in terms of cell quantity and quality, the level of iron storage and fibrosis. All malnutrition abnormalities and deficiencies in serum levels of the mentioned components and the percentage of cellular fractions of BM aspirates were corrected using supplement therapy before the cell infusion. The experimental group received IV infusions of autologous BM-derived MNCs and the control group received only autologous cell-free serum. For each individual a total of 20 ml of the cell suspension or cell-free serum was infused gradually. The cell infusion performed at the baseline. The patients were admitted and examined at Shariati Hospital (Mashhad, Iran) one day before cell infusion. 

The proposal of this study was reviewed and approved by the Ethics Committee of Mashhad University of Medical Sciences, and registered for clinical trial studies in Iranian Ministry of Health (MOH). The registration number is IRCT2014091919217N1. This study was conducted in accordance with the Declaration of Helsinki. All patients were provided with written informed consent.

### Cell preparation and transplantation 

The BM samples (140-200 ml/patient) were collected under local anesthesia and general sedation in an operating room under sterile conditions. All collected BM aspirates were filtered to remove any fat, bone, clot and other possible particles that could be collected in blood collection bags. The remaining MNCs were washed and counted and their viability was assessed using trypan blue dye exclusion method. The mean viability of the transplanted MNCs was more than (95 ± 3) % in all the infusions. The MNCs were suspended in autologous serum at the final volume of 20 ml. Finally, in each patient the general condition, vital signs and any transfusion-related reactions were monitored for six hours after cell infusion.

### Long term follow-up

A gastroenterologist examined the patients at baseline (0), 3, and 6 months post-infusion. Ablood analysis was requested at each visit as follows: complete blood count, serum albumin and total bilirubin, blood urea nitrogen, PT and INR. The MELD score was also measured accordingly. No adverse effects were detected during or after cell transfusion. 

### Statistical analysis

We have presented our data as mean ± SD. A two-way ANOVA followed by Sidak’s multiple comparisons test was performed using Prism graphpad version 6.00 for Mac OS X graphpad Software, La Jolla, California, USA. A P<0.05 was considered statistically significant. Furthermore, ANCOVA was used to evaluate the difference in means of control and experimental groups, considering time covariate effect. 

## Results

### The demographic data of the patients

We had initially recruited a total of 34 patients, however, 14 patients were excluded according to the inclusion/ exclusion criteria and ultimately 20 of them enrolled in the study (female/male ratio of experimental and control groups were 1/9 and 2/8, respectively). The mean age of the patients was 45.2 years (28-58 years old) and 46 years (21-62 years old) in control and experimental groups, respectively. The etiology of cirrhosis in four (20%) patients were autoimmune hepatitis (AIH), nine patients (45%) had suffered from viral hepatitis, five (25%) had an unknown origin, one (5%) had PSC and one (5%) had Wilson’s disease. The descriptive underlying etiologies of the disease and demographic data of the participating patients are listed in Table 1. All the patients with nutritional deficiencies received supplement therapy prior to enrollment in the study.

### The initial peripheral blood testing and bone marrow aspiration results

The peripheral blood tests of the patients were performed before BM aspiration and cell infusion. Hemoglobin concentration of the patients ranged between 12 to 17 g/ dl, but one male patient had a mild anemia (Hb<12 g/dl). The folate level (5 to 20 ng/ml) and the B12 level (59 to 895 pg/ml) were normal in all patients except for two individuals, who had folate levels less than 4.9 ng/ml and B12 levels less than 59 ng/ml. The transferrin saturation was normal in all patients (18-47%). The minimum concentration of ferritin was 12.8 ng/dl and its maximum concentration was 542 ng/dl. The normal range for ferritin starts at 12 ng/ml. 

BM analysis showed no high-grade fibrosis in the patients. Moreover, five individuals had mild to moderate megaloblastic changes, three patients had micronormoblastic changes and the remaining had normal cellular analysis reports.

The mean weight of patients was 67.5 ± 4.8 kg. The mean number of total nucleated cell counts (TNC) was (8.46 ± 2.56×103/µl) that (58.59 ± 7.834)% of them were polymorphonuclear cells (PMN) and (41.41 ± 7.834)% of them were MNCs. The mean number of transfused cells was (8.059 ± 2.539×10^6^ cells/kg). Table 2 represents the number of MNCs that were infused into the patients. 

**Table 1:** Descriptive underlying etiologies of the cirrhosis and demographic data of patients in experimental and control groups


		Experimental group			Control group	
Patients ID.	Age (Y)	Gender	Etiology	Age (Y)	Gender	Etiology

P1	30	Male	AIH	28	Male	AIH
P2	37	Male	Hepatitis B	45	Male	Cryptogenic
P3	56	Male	Hepatitis C	54	Female	Hepatitis B
P4	28	Female	AIH	44	Male	PSC
P5	21	Male	Cryptogenic	58	Male	Cryptogenic
P6	54	Male	Hepatitis C	39	Male	Hepatitis B
P7	62	Male	AIH	50	Female	Cryptogenic
P8	56	Male	Hepatitis B	47	Male	Hepatitis B
P9	58	Male	Cryptogenic	58	Male	Wilson
P10	58	Male	Hepatitis B	29	Male	Hepatitis B


PSC; Primary sclerosing cholangitis and AIH; Autoimmune hepatitis.

**Table 2:** The number of transfused MNC for patients in group 1


Patients ID.	TNC (10^3^/µl)	PMN (%)	MNC (%)	BW (kg)	Transfused MNC (10^6^ cells/kg)

P1	11.2	65.8	34.2	72	8.1
P2	12.6	56.3	43.7	67	12.3
P3	7.1	49.4	50.6	68	7.7
P4	9.7	63.5	36.5	65	8.3
P5	3.3	74.3	25.7	59	2.15
P6	8.4	56.5	43.5	67	8.05
P7	8.8	54.3	45.7	64	9.5
P8	6.9	61.1	38.9	75	7.2
P9	7.3	48.3	51.7	73	7.8
P10	9.3	56.4	43.6	65	9.49


TNC; Total nucleated cell, PMN; Polymorphonuclear cell, MNC; Mononuclear cell, and BW; Body weight.

### Model of end-stage liver disease score 

**Fig.1:**
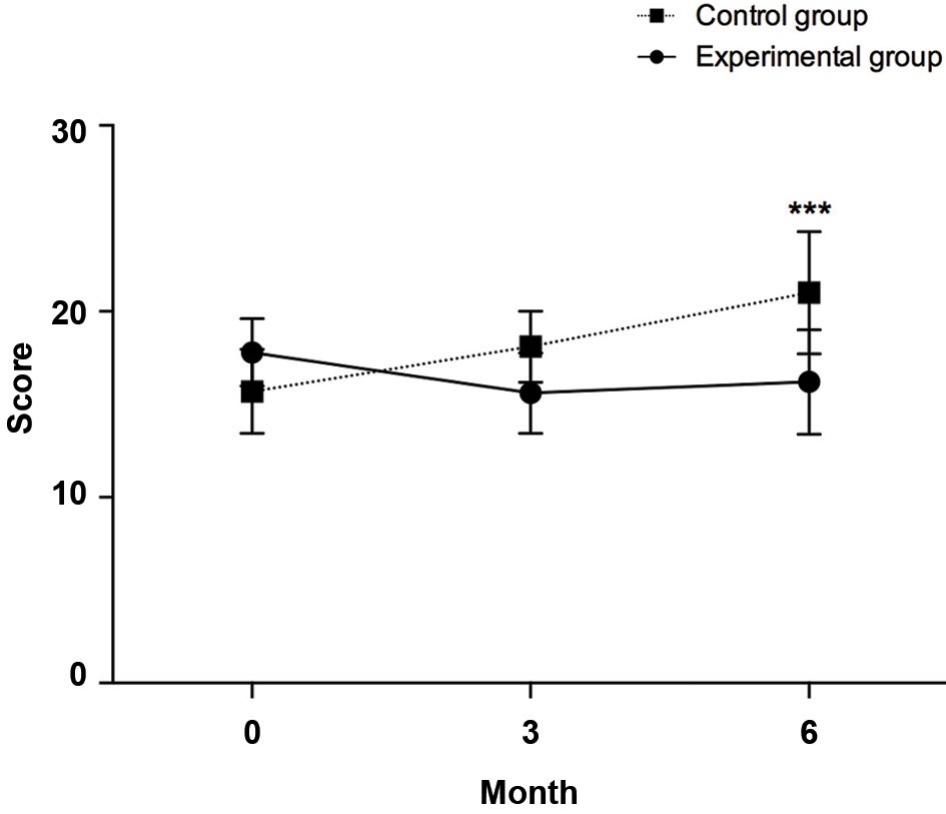
Changes in MELD scores between control and experimental groups atdifferent time points (baseline, three and six months post-transplantation). ***; P≤0.001.

At 6 months post-cell transfusion the MELD score decreased in the experimental group to 16.2 ± 2.82, which is statistically significant compared to the control group 21 ± 3.29 (P=0.0001). In the experimental group, there was no significant difference between 0 (baseline) (17.80 ± 1.81), 3 (15.60 ± 2.17) and 6 months (16.20 ± 2.82) after transplantation (Fig .1), whereas, compared to the control group, the MELD score improved significantly 6 months post-cell infusion. Next, the influence of time as the covariate on independent MELD score in control and experimental groups were checked by ANCOVA. There was a significant difference in MELDI mean score [F (4,54)=5.272, P=0.001] between the cases and controls, whilst adjusting for time. It can be seen that for cases and controls the effect size is small (0.281). 

### International normalized ratio

**Fig.2:**
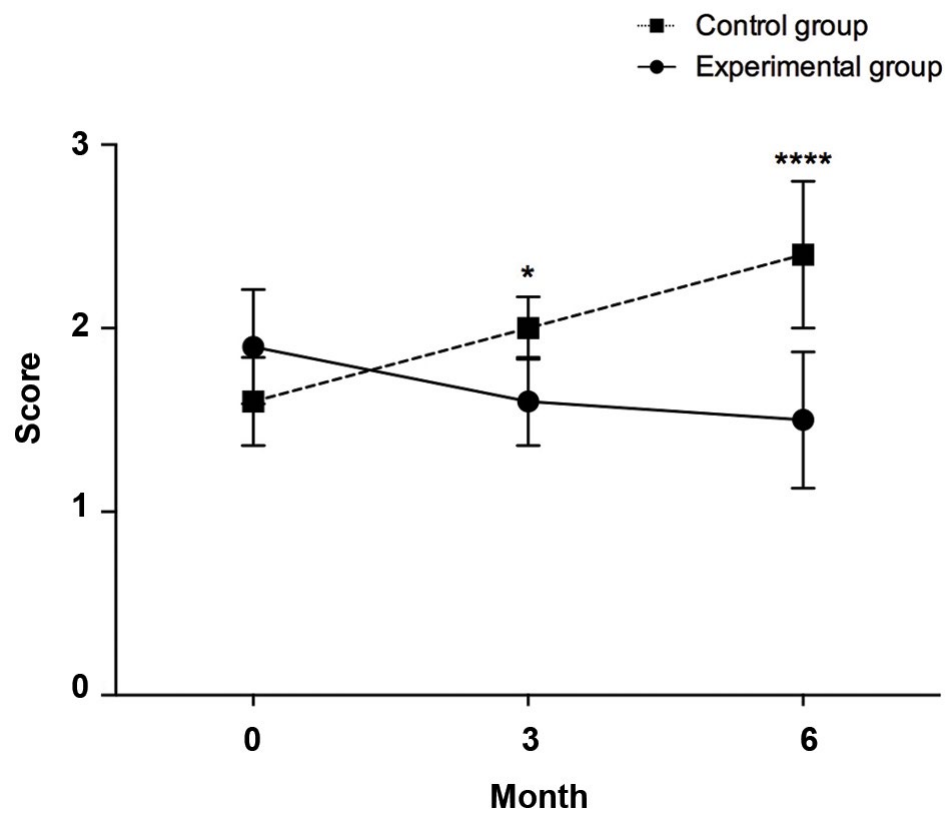
Changes in international normalized ratio (INR) between the control and experimental groups at different time points [0 (baseline), 3 and 6 months post-transplant]. *; P=0.05 and ****; P≤0.0001.

The INR score of the control group increased from the baseline to 2.0 ± 0.17 and 2.4 ± 0.4 in 3 and 6 month post-transplantation, respectively. However, the score in the experimental group decreased slightly during the 6 months, which was statistically significant in both 3 (1.6 ± 0.24, P=0.012) and 6 (1.5 ± 0.37, P<0.0001) months post-cell infusion (Fig .2). In the experimental group, the INR score at 6 months (1.5 ± 0.37) was even significantly lower compared to its baseline level (1.9 ± 0.31, P=0.01). Next, the influence of time as the covariate on INR in control and experimental groups were checked by ANCOVA. There wasn’t a significant difference in INR mean score [F (4,54)=0.989, P=0.422] between the cases and controls, whilst adjusting for time. 

### Albumin 

**Fig.3:**
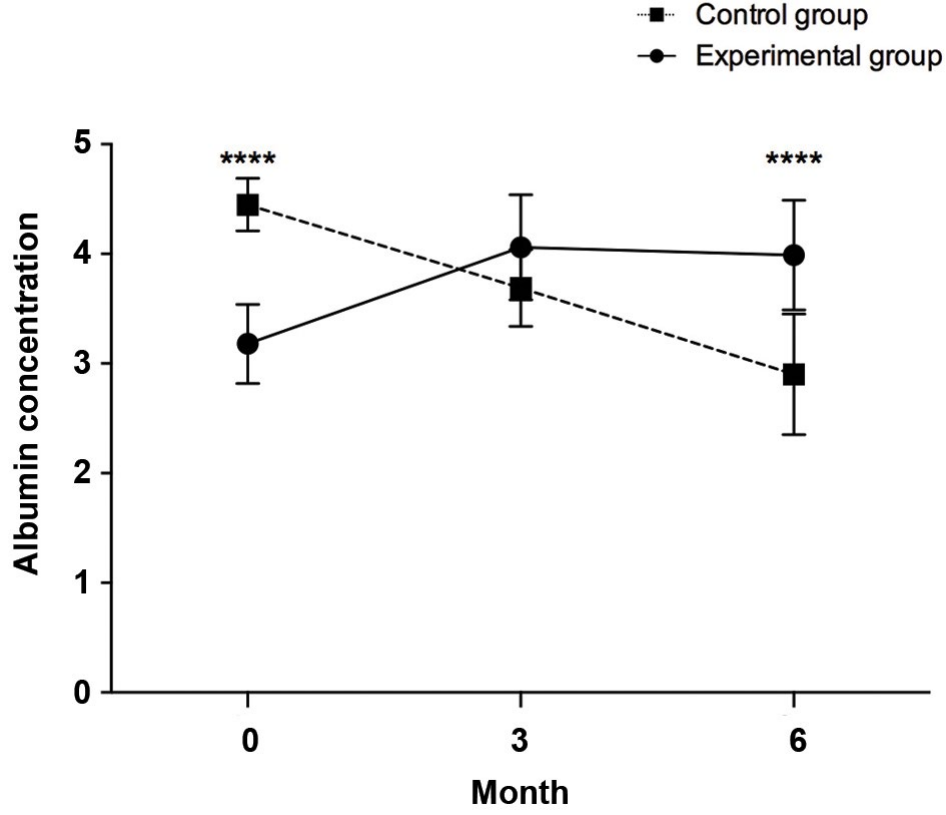
Changes in albumin levels between the control and experimental groups at different time points [0 (baseline), 3, and 6 months post- transplant]. ****; P≤0.0001.

Serum albumin levels of the patients in control and experimental groups were different at baseline (control, 4.45 ± 0.25 vs. experimental, 3.18 ± 0.36), which was statistically significant (P<0.0001) and this lowered the power of study. As shown in Figure 3, at 6 months after the treatment, the average serum albumin level of the experimental group increased significantly compared to the control group (3.99 ± 0.50 vs. 2.90 ± 0.55, P<0.0001). Next, the influence of time as the covariate on independent Serum albumin level in control and experimental groups were checked by ANCOVA. There was a significant difference in Albumin mean Level [F (4,54)=25.454, P<0.0001] between the cases and controls, whilst adjusting for time. It can be seen that for cases and controls the effect size is moderate (0.653).

**Fig.4:**
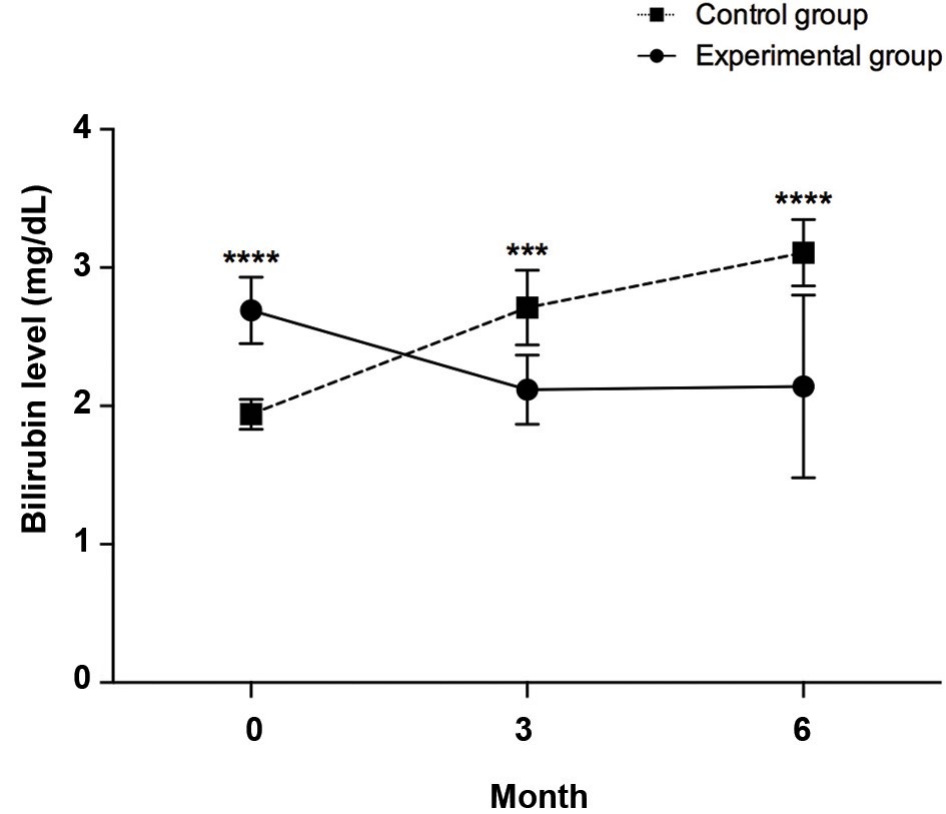
Changes in bilirubin between control and experimental groups at different time points (0, 3 and 6 months post-transplantation). ***; P=0.001 and ****; P≤0.0001.

The serum level of albumin in the experimental group increased at 3 months post-transplantation (4.06 ± 0.48, P<0.0001), but slightly decreased by the 6 months time point (3.99 ± 0.50, P=0.0003). At both time points, however, the overall increases were statistically significant in comparison with the baseline level (3.18 ± 0.36, Fig .4).

### Bilirubin 

Serum bilirubin of the control group patients increased sharply and reached up to 3.11 ± 0.24 at 6 months after serum (placebo) infusion, whereas in the experimental group we observed a significant decrease in bilirubin level (2.12 ± 0.25, P=0.0009), 3 months after cell infusion. The significant decrease in bilirubin was observed at 6 months (2.14 ± 0.66, P<0.0001) after cell infusion, which was statistically significant compared to the control group.

The decrease in bilirubin level in the experimental group at 3 (2.12 ± 0.25, P=0.0014) and 6 (2.14 ± 0.66, P=0.002) months post-transplantation were also statistically significant compared to the baseline levels (2.69 ± 0.24, Fig .4). Next, the influence of time as the covariate on independent serum bilirubin level in control and experimental groups were checked by ANCOVA. There was a significant difference in bilirubin mean Level [F (4,54)=22.464, P<0.0001] between the cases and controls, whilst adjusting for time. It can be seen that for cases and controls the effect size is moderate (0.625). 

## Discussion

Based on this study, after nutritional supplement treatment, hematopoietic stem cells transplantation could transiently improve MELD score, serum albumin level, INR score, as well as bilirubin level in cirrhotic patients. BM stem cell infusion results in considerable improvements in different disorders in basic and clinical studies (9, 14-16), particularly in clinical trials for liver cirrhosis in human patients (17-29). 

Due to the short follow up period after transplantation (6 months), the efficacy of MNC infusion for an extended time is not clear yet. A transient improvement in MELD score was observed in the experimental group, but it was not statistically significant, at 6 months compared to the previous time point (3 months) after cell infusion. Nonetheless, in some previous studies, the follow up period after stem cell transplantation in cirrhotic patients has been up to 30 months with improvements in liver function tests (21). Therefore, our MELD data may have been affected by the shorter follow up period in our study.

In another previous study, the authors observed that MNC transplantation resulted in transient improvements in liver function, but it did not lead to a complete reversal from an abnormal condition into normal liver physiologic conditions (21). Therefore, given the fact that the mortality rate in patients in organ waiting lists is high, BM-derived MNCs may potentially give the cirrhotic patients a higher chance of survival while waiting for a matching liver for OLT (30).

Based on the results of the BM study and the peripheral blood testing data in cirrhotic patients, there were obvious discordances between the two. Presumably, BM is affected by nutritional deficiency in earlier stages of cirrhosis.

Stem cell therapy has had a growing progress in many disabling diseases, worldwide. Nowadays, different hematologic and non-hematologic disorders may be treated using stem cell transplantation. However, there are many limitations and doubts in terms of effectiveness of stem cell transplantation in liver cirrhosis. Scientists are now working on novel therapies to overcome these challenges.

## Conclusion

We have shown that transplantation of BM-derived stem cells, which is a relatively safe procedure, transiently improves some crucial parameters in cirrhotic patients after correcting nutritional anomalies. However, to have a better and clearer conclusion, there need to be more basic and clinical studies and randomized clinical trials with a higher number of subjects with perhaps uniform etiologies. 
